# Mineral Springs and the New Element, Niton

**Published:** 1913-01

**Authors:** 


MINERAL SPRINGS AND THE NEW ELEMENT, NITON. Dr. L. K. Hirshberg, of Johns Hopkins, (*Scientific American*, April 20, 1912). Thomas, Ramsay, Crookes and Rutherford, found traces of radium in the waters of many mineral springs Then followed the discovery by Sir William Ramsay, not only of the fact that the wonderful properties of radium were due to the gas niton, of which it contains seventy-five per cent, but also that the curative value of the spring at Bath, England, was due to the niton its waters contained.

We are thus furnished with an explanation for a phenomenon which has given rise to considerable speculation, viz., the fact that mineral waters that are prepared artificially, even though the formula of the bona fide spring is reproduced qualitatively and quantitatively with the utmost care, fail to produce the same effects as the waters of the original spring, especially when used at the spring itself. Even the greater activity of freshly drawn waters may find its explanation in the fact that while niton, like helium and argon, is practically inert and forms no chemical salts or other compounds, its existence is but temporary, lasting a little less than four days—3.86 days, to be exact—when it becomes transformed into something new. Whether this change actually occurs in mineral waters, however, we are not told.

Sir Walliam Ramsay, has further shown that, as is the case with other gases, niton is compressible, a simple compression at room temperature sufficing to convert it into a-liquid. Moreover, when this liquid is pressed into a silica glass tube having a very fine bore, it becames solidified into a microscopic rod of transparent icelike material. Though its transparency renders it invisible, it emits a brilliant light.

